# Effects of Cytoplasmic Sterility on Roots and Yield of Nitrogen Sources in Rice

**DOI:** 10.3390/plants14050820

**Published:** 2025-03-06

**Authors:** Rong Liu, Qin Wang, Xiyuan Wang, Shengmin Yan, Guotao Yang, Peng Ma, Yungao Hu

**Affiliations:** 1 College of Life Science and Engineering, Southwest University of Science and Technology, Mianyang 621010, China; liurong0104@swust.edu.cn (R.L.); wangqin17022@163.com (Q.W.); xiyuan03030505@163.com (X.W.); yangguotao2377893@163.com (G.Y.); 2 Zigong Academy of Agricultural Sciences, Zigong 643000, China; yan_shengmin@126.com

**Keywords:** hybrid rice, WA cytoplasm, JW cytoplasm, nitrogen form, uptake rate

## Abstract

Rice is an important food crop, acting as the staple food for more than 50% of the global population. We selected seedlings (two sterile male lines: WA803A and JW803A) that had different cytoplasmic but the same nuclear composition and were heterogeneous. The maintainer line 803B was also used. We aimed to study their nitrogen uptake rate in different concentrations of NH_4_^+^ and NO_3_^−^ and explore the differences in nitrogen uptake efficiency between different cytoplasmic genes. The results showed a significant difference in the nitrogen uptake rate for different seedlings. With ammonium nutrition, the nitrogen uptake efficiency of the JW cytoplasm was significantly higher than that of the WA cytoplasm. In low concentrations of ammonium nitrogen, the JW cytoplasm had an additive effect to the nuclear gene regulation of ammonium uptake. The JW cytoplasm’s ammonium nitrogen absorption effect on nuclear gene regulation was higher than that of the WA cytoplasm. The effect of the WA and JW cytoplasms on the nitrate uptake rate was not significant, and the nuclear gene regulation of both cytoplasms was reduced by absorbing nitrate. Under nitrogen deficiency conditions, the material output and conversion rate of the JW-type cytoplasmic hybrid rice combination was relatively high, significantly higher than those of other cytoplasmic combinations. Under medium nitrogen conditions, the material output and conversion rate of the (N2) W-type hybrid rice combination were significantly higher than those of the other cytoplasmic combinations. The yield of JW-type rice first increased and then decreased with the increase in the nitrogen application rate and was highest, 8195.55 kg/hm^2^, under the N2 treatment.

## 1. Introduction

Since 1985, China has been the world’s largest consumer of nitrogen fertilizers. Compared with the world average, the amount of nitrogen fertilizer used in rice fields in China and its proportion amongst the fertilizers used are relatively high. For example, currently, the nitrogen fertilizer used in rice production in China accounts for approximately 37% of the total amount of nitrogen fertilizer used in world rice production [1]. However, the nitrogen fertilizer utilization rate in China is approximately 30%, far below the world average [2,3]. Additionally, the amount of nitrogen applied to rice fields in China generally exceeds 180 kg/hm^2^, much higher than the world average of 105 kg/hm^2^ [4]. In the southern rice-growing areas of China, 0.9–1.25 kg of nitrogen is necessary for every 50 kg of rice grain produced, and the amount of nitrogen applied to rice in a single season has exceeded 350 kg/hm^2^, with more than 56% of the nitrogen fertilizer used applied as basal tiller fertilizer [5]. Excessive nitrogen fertilizer application and unscientific fertilization methods not only increase production costs but also cause a sharp decrease in nitrogen fertilizer utilization (nitrogen use efficiency is the proportion of nitrogen in the fertilizer that is absorbed and utilized by the crop) in rice fields, leading to wasted resources and increased environmental pollution [6,7]. Under the premise of ensuring improved yield, selecting nitrogen-efficient rice varieties not only reduces the application of nitrogen fertilizer to fields but also reduces the risk of environmental nitrate emissions [8]. Therefore, breeding nitrogen-efficient rice varieties, improving nitrogen utilization efficiency, and reducing nitrogen fertilizer application rates are considered feasible [9] and have become hot topics in rice breeding worldwide [10]. Between indica and japonica rice, and hybrid and conventional rice, different genotypes of the same type of rice [11] show significant differences in their nitrogen absorption and utilization, and indica rice is more responsive to nitrogen than japonica rice [12,13]. 

The main types of nitrogen absorbed and utilized by rice are NH_4_^+^ and NO_3_^−^. The nitrogen absorbed by rice roots differs from that exchanged with [H^+^] to absorb NH_4_^+^ during other processes that are ongoing in the cytoplasm and chromosomes in rice roots, in which nitrogen from the environment is restored and translated to nitrogenous organic compounds, such as proteins [14,15]. At the molecular level, the transmembrane transport and translocation of inter-root nitrogen into rice require the assistance of specific transporter proteins, with the NO_3_^−^ transporter family (nitrate transporter, NRT) and the NH_4_^+^ transporter family (ammonium transporter, AMT) being responsible for the uptake and translocation of NO_3_^−^ and NH_4_^+^, respectively [16,17]. The synthesis and distribution of different types of transporter proteins have obvious temporal and spatial specificities and are significantly affected by external nitrogen patterns and nitrogen supply levels, unlike genes. Therefore, the root is a key organ for the absorption and transport of nitrogen in rice, and the effect of nitrogen on the root system’s growth and nitrogen distribution is obvious in different mediums [18]. Moderate nitrogen deficiency inhibits the growth of the overground parts of rice and promotes root growth, and a serious nitrogen deficiency can inhibit plant growth altogether [19]. The root mechanisms leading to the differences in the efficiency of nitrogen absorption of different genotypes of rice have been reported in depth [20]. Additionally, some studies have reported that the combined ability of rice to absorb and utilize nitrogen is obviously different in different cytoplasmic male sterile lines [21,22,23]. (Male sterility [MS] is widely found in higher plants in nature and refers to the genetic phenomenon of female gametes developing normally, male gametes developing abnormally, and the female gametes being fertilized normally with foreign pollen. MS mainly pertains to cytoplasmic MS and genetic MS. Cytoplasmic MS, also known as nucleoplasmic MS, is a maternal genetic phenomenon that results in abnormal fertilization and fruiting due to pollen failure and is jointly regulated by mitochondrial sterility genes and restorer of fertility (Rf) genes.) Nonetheless, the absorptive capacity of sterile lines toward different nitrogen sources has not been reported.

In this paper, we investigated the differences in the uptake capacity of the roots of two commonly used homokaryotic heteroplasmically and cytoplasmically sterile lines in different concentrations of nitrate and ammonia nitrogen to clarify the uptake characteristics of genetically similar but cytoplasmically different rice with respect to different nitrogen sources.

## 2. Materials and Methods

### 2.1. Experimental Material

**Figure 1:**
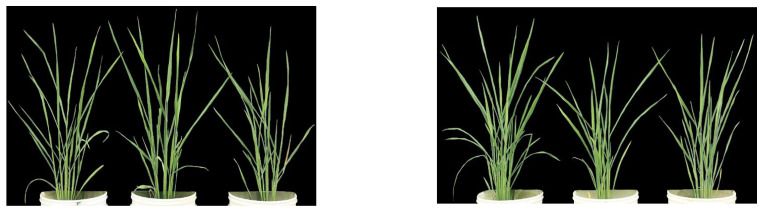
Response of different homo nuclear hetero sterile lines to nitrogen fertilizer treatment (N1:N2:N3).

We selected two different cytoplasmically sterile lines: a JW type and a wild abortion type. A set of isonuclear alloplasmic male sterile lines, named JW803A and WA803A (with nuclear exchange 19th generation by the spring of 2023), was transferred using high-quality maintainer line 803B with high yield and high nitrogen uptake efficiency; maintainer line 803B was used as the control. These two homozygous heterozygous sterile lines were tested using different nitrogen treatments, N1, N2, and N3, in the pre-treatment experiments, with nitrogen application levels of 120, 180, and 240 kg/hm^2^, respectively, and JW803A showed strong tolerance to low nitrogen under medium and low nitrogen conditions (Figure 1).

### 2.2. Experimental Methods

After sterilization of 0.1% mercuric chloride, the seeds of the sterile line and the maintainer line were soaked for 48 h with an interval at 25 °C. These seeds were then sowed in a germination box covered with a nylon net after accelerating germination for 1 d at 30 °C. They grew into seedings in an illumination incubator for 12 h per day at 28 °C. The seedlings were cultivated with International Rice Research Institute nutrient solution (formulation source, International Rice Research Institute; this article is cited as Coolaber Science & Technology Co., Ltd., Beijing, China, www.coolaber.com, accessed on 16 February 2025) after a single leaf appeared. The interval time of aeration was approximately 2 h. The culture solution was replaced every 5 d until the two-leaf stage.

#### 2.2.1. Absorption of NH_4_^+^ in Rice

The NH_4_^+^ absorbent solutions were prepared with (NH_4_)_2_SO_4_, the concentrations of which were 0.05, 0.10, 0.20, 0.40, 0.60, 1.00, 1.40, 1.80, and 2.20 mmol/L, and the concentrations of other nutrient elements were invariant (NH_4_NO_3_ 144.3mg/L, NaH_2_PO_4_ 50.4 mg/L, CaCl_2_ 158.2 mg/L, and MgSO_4_•7H_2_O mg/L). Before the absorption test, 2 d of nitrogen starvation resulted in the rice having the status of nitrogen starvation. The supporting electrolyte was CaSO_4_ with 0.2 mmol/L, and the pH of the solutions was 6.0–6.2. Three strong rice seedlings were sampled as a measurement unit, the whole roots of which were submerged in a glass tube with 20 mL NH_4_^+^ absorbent solution; 2 h were used to assimilate them to a temperature of 29 ± 1 °C and an illumination intensity of 162 umol/s/m^2^. The roots were weighed and the NH_4_^+^ content in the nutrient solution was tested before and after absorption. This experiment was repeated thrice. The unit dry root focused on the nitrogen net uptake per unit time, which was the nitrogen net uptake rate in the rice roots, and was calculated using the variation in NH_4_^+^ concentration before and after absorption (measuring the content of NH_4_^+^ by indophenol blue spectrophotometry [24]).

#### 2.2.2. Absorption of NO_3_^−^ in Rice

Except for the chemical replaced with KNO_3_, other methods were the same as in Section 2.2.1 (measuring the content of NO_3_^−^ by ultraviolet spectroscopy). The net absorption rate was calculated using the following equation:

(1) $$V=\frac{C_{1}\times m_{1}-C_{2}\times m_{2}}{12\times m_{3}}$$

Note: *V* is the net absorption rate; *C*_1_ is the concentration of NO_3_^−^ or NH_4_^+^ before absorption; *C*_2_ is that after absorption; *m*_1_ is the weight of the seedlings, glass tubes, and solutions before absorption; *m*_2_ is that after absorption; and *m*_3_ is the fresh weight of the roots.

#### 2.2.3. Determination of Dry Matter and Nitrogen Content Indicators

During the panicle initiation stage of rice growth, three representative rice plants were selected, killed using 105 °C heat for 1 h, and dried at 80 °C until a constant weight was achieved. The dried straw was divided into four parts: roots, stems/sheaths, leaves, and panicles. After measuring the dry weights, the samples were pulverized and passed through a 100-mesh sieve. The Kjeldahl method was used to determine the nitrogen content of each part after digestion using sulfuric acid and hydrogen peroxide [25].

### 2.3. Experimental Design

The experiment was conducted in the experimental base of Rice Research Institute of Mianyang Southwest University of Science and Technology in 2022 and 2023. The soil was loam. The basic fertility of the experimental field was 1.98 g/kg total nitrogen, 80.3 mg/kg available nitrogen, 43.3 mg/kg available phosphorus, and 76.2 mg/kg available potassium. The positioning test of different fertilizer treatments began in 2017. The experiment was designed with four nitrogen levels, N0, N1, N2, and N4, and the pure nitrogen application rates were 0, 90, 180, and 270 kg/hm^2^, respectively; a ratio of 5:3:2 was used for the base fertilizer, tillering fertilizer, and panicle fertilizer. The amount of phosphate fertilizer (P_2_O_5_) was 60 kg/ha, which was applied once as base fertilizer. The amount of potassium fertilizer (K_2_O) was 120 kg/ha, which was applied as base fertilizer and panicle fertilizer in a ratio of 1:1. The sowing date of the tested rice varieties in 2022 and 2023 was 10 April, and the planting date was May 12. The test plot was 7 m long and 7 m wide. The planting area of each plot was 3.5 × 7 m; 20 rows were planted, with 20 plants per row; and the planting specifications were 33.3 cm × 16.7 cm. Each nitrogen fertilizer treatment was set up with three replicates and a total of 12 plots. Each interval was separated by a 50 cm thick 30 cm cement ridge to prevent fertilizer and water loss.

### 2.4. Data Analysis

Analyses of variance (ANOVA) and least significant difference (LSD) tests were used to compare data using SPSS v23 (Chinese version v22.0.0.0) (Statistical Product and ServiceSolutions Inc., Chicago, IL, USA), with a significance threshold of *p* < 0.05. Figures were constructed using Origin Pro 2023 (OriginLab, Northampton, MA, USA).

## 3. Results

### 3.1. Significance Analysis of Different Factors in Nitrogen Absorption Efficiency

Table 1 shows the analysis of variance of genotypes and solution concentrations under different morphological nitrogen conditions. Highly significant differences existed in the nitrogen absorption efficiency of different genotypes and at different concentrations of nitrogen. Furthermore, the interaction between gene types and nitrogen concentrations was highly significant. The maximum in the variance of nitrogen solution concentration indicates that nitrogen concentrations were the dominant factor, which affected nitrogen absorption efficiency.

### 3.2. Effect of Different Forms and Different Solution Concentrations of Nitrogen on Nitrogen Absorption Efficiency in Rice

#### 3.2.1. Ammonium Nitrogen

**Figure 2:**
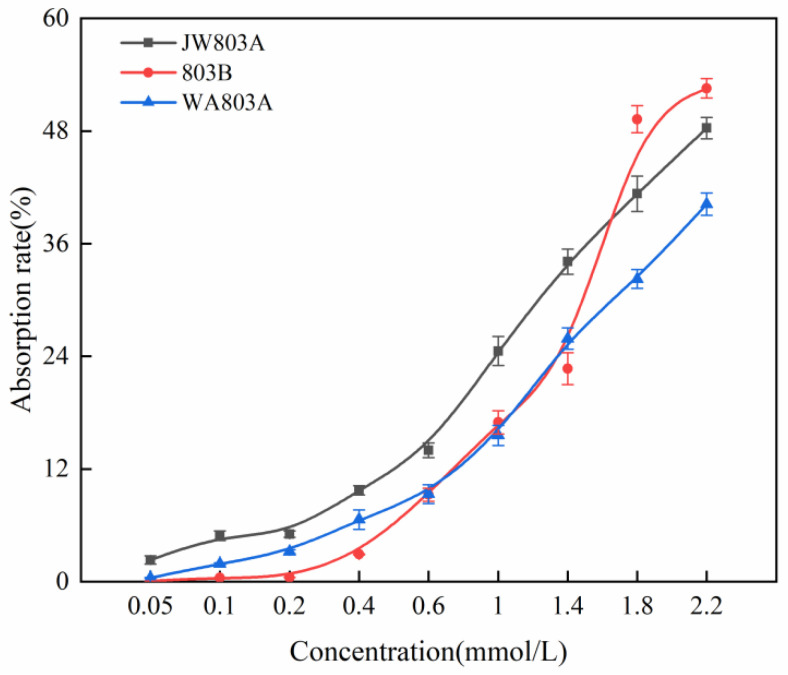
The absorption rate of ammonium nitrogen in WA803A, 803B, and JW803A. Note: error bars represent standard errors; each value represents the average of three repetitions of the test.

We compared the effect of different concentrations of ammonium on absorption efficiency in rice (Figure 2). The absorption rate curve of ammonium nitrogen in WA803A and JW803A, which were homo nuclear alloplasmic male sterile lines, had the same tendency: the absorption rate increased as the concentration increased. Moreover, the absorption rate curve of maintainer line 803B was similar to that of WA803A and JW803A, but the linear relation between variation and concentration was lower. The absorption rate of 803B was lower than that of WA803A and JW803A in the lower concentration of ammonium nitrogen (<0.6 mmol/L). Subsequently, the absorption rate of 803B exceeded that of WA803A; the absorption rate was less than that of WA803A and JW803A (1.1 mmol/L). The absorption rate of 803B was greater than that of WA803A and JW803A when the concentration increased to 1.5 mmol/L.

#### 3.2.2. Nitrate Nitrogen

**Figure 3:**
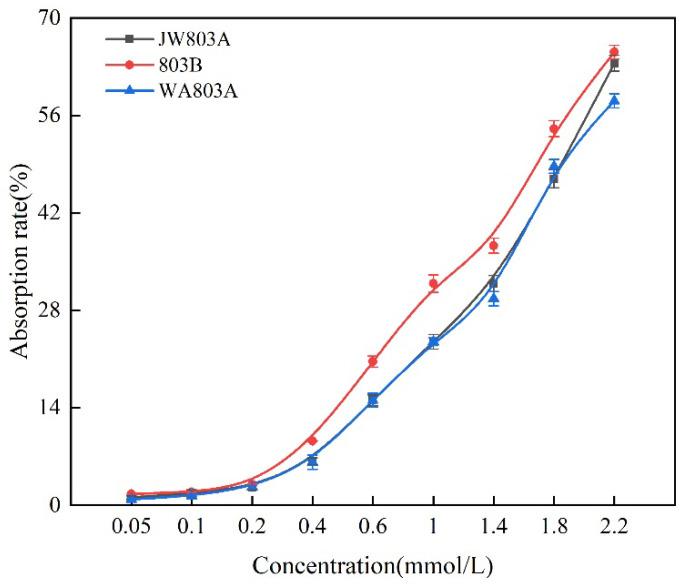
The absorption rate of nitrate nitrogen of WA803A, 803B, and JW803A. Note: error bars represent standard errors; each value represents the average of three repetitions of the test.

We measured the effect of different concentrations of nitrate on absorption efficiency in rice (Figure 3). The absorption rate curve of nitrate nitrogen of WA803A and JW803A had the same tendency as that of the absorption rate increase with the increase in concentration. The absorption efficiency of the two male sterile lines was the same for nitrate nitrogen until the concentration of 1 mmol/L and was different subsequently. The absorption efficiencies were higher at a concentration of 1–1.6 mmol/L and were lower than that of WA803A. When the concentration was 2 mmol/L, they were higher than that of WA803A and then gradually became near to that of WA803A.

### 3.3. Effect of Nitrate Uptake Efficiency of Different Genotypes

Three nitrate concentrations, 0.4, 1, and 1.8 mmol/L, were selected to analyze the significance of the differences in nitrate uptake efficiency of different genotypes.

#### 3.3.1. Ammonium Nitrogen

**Figure 4:**
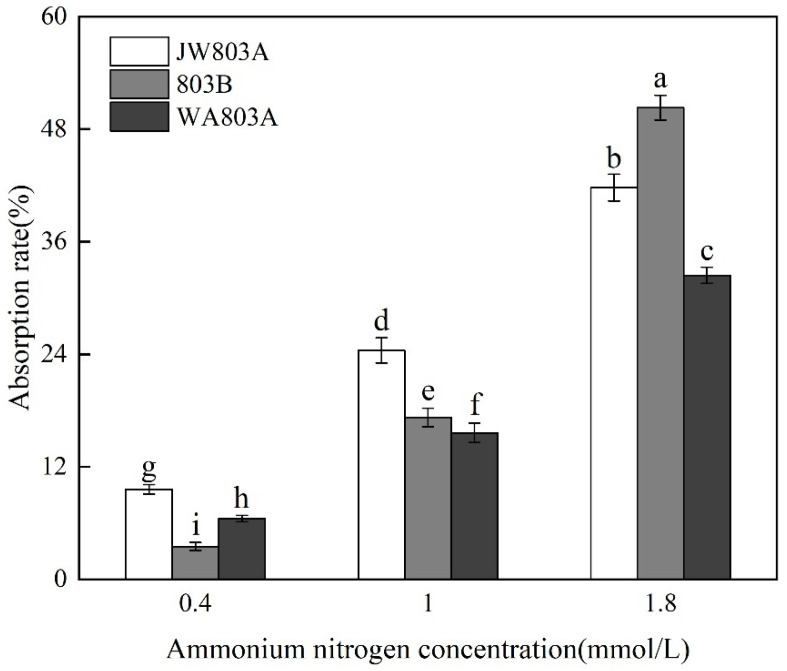
Ammonium nitrogen absorption efficiency of different cytoplasms. Note: values followed by different letters within a row indicate differences (*p* < 0.05) between different parameters; error bars represent standard errors; each value represents the average of three repetitions of the test.

The nitrogen absorption rates were significantly different in WA803A, JW803A, and 803B (Figure 4). At the low concentration, the ammonium nitrogen absorption rate in JW80A was significantly higher than that in WA803A and 803B; that in WA803A was significantly higher than that in 803B. At the middle concentration, the ammonium nitrogen absorption rate in JW803A was significantly higher than that in WA803A and 803B; in 803B, it was significantly higher than that in WA803A. At the high concentration, the ammonium nitrogen absorption rate in 803B was significantly higher than that in the two isonuclear alloplasmic male sterile lines; that in JW803A was always higher than that in WA803A. The ammonium nitrogen absorption rate in the same rice material showed significant differences at different concentrations of ammonium nitrogen; at the high concentration, it was significantly higher than that at the low concentration.

#### 3.3.2. Nitrate Nitrogen

**Figure 5:**
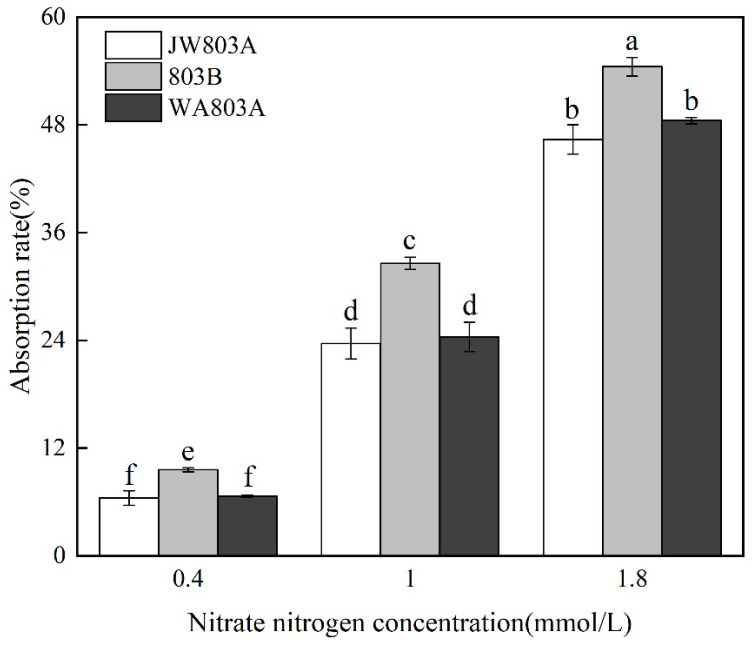
Nitrate nitrogen uptake efficiency of different cytoplasms. Note: values followed by different letters within a row indicate differences (*p* < 0.05) between different parameters; error bars represent standard errors; each value represents the average of three repetitions of the test.

The nitrate nitrogen uptake rate was the same and had no significant difference in two different cytoplasm genotypes, WA803A and JW803A, at the same concentration of nitrate nitrogen (Figure 5). However, the uptake rate in maintainer line 803B was significantly higher than that in WA803A and JW803A both at high concentrations and low concentrations. The uptake rate in the same rice material had significant differences at different concentrations of ammonium nitrogen. The uptake rate at high concentrations was higher than that at low concentrations.

### 3.4. Influence of Cytoplasm Types on Nuclear Effect Under the Condition of Ammonium Nitrogen

The results (Table 2) indicated that compared with that in 803B, the uptake rate in WA803A was low at three concentrations of ammonium nitrogen, namely, low concentration (0.05 mmol/L), middle concentration (0.6–1.0 mmol/L), and high concentration (1.8 mmol/L); high at other concentrations; and more than 200% than in 803B, especially at low concentrations (0.10–0.20 mmol/L). The uptake rate in JW803A was lower than in 803B at the high concentration of ammonium nitrogen (1.80 mmol/L) and higher than that in 803B at other concentrations. Additionally, the absorption rate in JW803A was 100% higher than that in 803B at low concentrations (0.05–0.40 mmol/L) and 500% higher than in 803B at the same low concentrations as WA803A. The regulation ability of ammoniacal nitrogen absorption by nuclear genes in JW803A was higher than that in WA803A (within 1.40 mmol/L). The nuclear effect of ammonium nitrogen absorption regulated by the JW cytoplasm was superior to that of the WA cytoplasm and had a positive effect under 1.4 mmol/L.

### 3.5. Influence of Cytoplasm Types on Nuclear Effect Under the Condition of Aitrate Nitrogen

As shown in Table 3, the absorption efficiency of nitrate nitrogen of two different cytoplasm sterile lines, WA803A and JW803A, was far below that of 803B and was higher than that of 803B only at the concentration of 0.1 mmol/L nitrate nitrogen. Additionally, the difference in absorption efficiency of nitrate nitrogen between WA803A and JW803A was small. Therefore, two different cytoplasms of the WA type and JW type had negative effects on controlling the absorption of nitrate nitrogen by nuclear genes.

### 3.6. Effects of Cytoplasm on Dry Matter Accumulation and Nitrogen Accumulation in Rice Under Different Nitrogen Fertilizer Treatments

**Figure 6:**
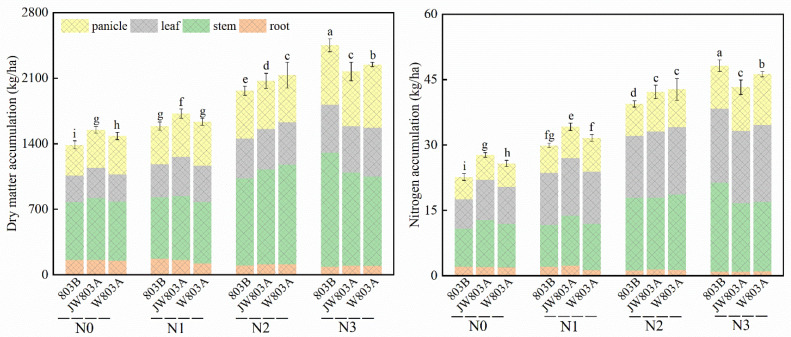
The effect of cytoplasmic male sterility on the accumulation of dry matter and nitrogen. Note: N1, N2, and N3 presented nitrogen application levels of 120, 180, and 240 kg/hm^2^; values followed by different letters within a row indicate differences (*p* < 0.05) between different parameters; each value represents the average of three repetitions of the test.

Significant differences existed in the total dry matter accumulation among WA803A, JW803A, and 803B during the rice panicle initiation stage (Figure 6). The analysis of the dry weights of different plant parts at this stage demonstrated that compared with the N0 treatment, all other nitrogen treatments showed a significant increase in dry weight. The dry matter accumulation in WA803A, JW803A, and 803B increased with increasing nitrogen application, and the dry weight of the roots decreased with increasing nitrogen levels. WA803A and JW803A had significantly higher total dry matter accumulation than 803B under the N0, N1, and N2 treatments. Under the N3 treatment, the dry matter of 803B was mainly concentrated in the stem/sheath, resulting in the highest total dry matter content for this cytoplasm type. Regarding the total nitrogen content in rice plants during the panicle initiation stage, WA803A, JW803A, and 803B showed the same trend as in dry matter accumulation, with an increase in total nitrogen content with increasing nitrogen application (Figure 6). With increasing nitrogen levels, the nitrogen content in the roots decreased, and the nitrogen content in the stem/sheath and leaves increased, leading to an overall increase in total nitrogen uptake by the rice plants. WA803A and JW803A had significant differences compared with 803B under the N0 and N1 treatments, with both cytoplasm types having higher total nitrogen content. Under the N3 treatment, the nitrogen content of 803B was mainly concentrated in the stem/sheath and leaves, resulting in the highest total nitrogen content for this cytoplasm type.

### 3.7. Response of Nutrient Organ Matter Export and Transport to Nitrogen Fertilizer Regulation 

The high yield of rice depends on the dry matter accumulation process of rice and is closely related to the transport of dry matter before and after flowering. The large amount of dry matter transported to the panicle after heading is the key to high yield and super high yield of rice. The material output rate of vegetative organs and stem/sheath of hybrid rice decreased first and then increased with the increase in nitrogen fertilizer application rate. Moreover, the material conversion rate showed the same trend (Table 4). The material output rate and conversion rate of each part were the lowest at the N2 level. In 2022 and 2023, the average material output rate of each combination of vegetative organs under this nitrogen application condition was 13.35% and 10.96%, respectively, and the conversion rate was 9.95% and 8.05%, respectively. The difference in the material output and conversion rate of vegetative organs in different years was small under nitrogen deficiency, and the difference between years under medium nitrogen and high nitrogen treatment was large. Extreme significant differences were observed in the output and conversion efficiency of substances under different nitrogen fertilizer levels. Under nitrogen deficiency conditions, the material output and conversion rate of the JW-type cytoplasmic hybrid rice combination was relatively high, significantly higher than other cytoplasmic combinations. Under the condition of medium nitrogen, the material output and conversion rate of the (N2) W-type hybrid rice combination were significantly higher than those of other cytoplasmic combinations. Under high nitrogen conditions, the material output and conversion rate of (N3) W-type hybrid rice combinations were relatively high. With the increase in positioning test years, some differences were observed in the material output and conversion rate between cytoplasm under different nitrogen fertilizer levels; however, the material output and conversion rate of JW-type cytoplasmic hybrid rice combinations remained at a relatively high level under nitrogen deficiency conditions. Additionally, the material output and conversion rate of hybrid rice were different between cytoplasms under nitrogen deficiency. In 2023, the coefficient of variation in the difference between the output and conversion rates of different cytoplasmic substances under high nitrogen conditions increased, which may be related to the difference in the response of cytoplasm to high nitrogen under excessive nitrogen residue conditions.

### 3.8. Differences in Yield Traits Under Different Nitrogen Fertilizer Levels 

To clarify the differences in the response of cytoplasm to different nitrogen fertilizer levels in yield traits, we compared the differences in yield traits between cytoplasms under different nitrogen fertilizer levels (Table 5). Under different nitrogen fertilizer conditions, the 1000-grain weight of hybrid rice increased first and then decreased with the increase in nitrogen application rate. The seed setting rate and grain setting rate decreased with the increase in nitrogen fertilizer application rate. The number of effective panicles increased with the increase in nitrogen fertilizer application rate. Among them, the number of effective panicles varied most with nitrogen fertilizer, and the average effective panicles under high nitrogen conditions increased by 40.33% and 5882 %, respectively, compared with no nitrogen treatment. Significant differences were observed in the number of grains per panicle among the cytoplasms under different nitrogen fertilizer conditions, and the difference of 1000-grain weight among the cytoplasms under N0 treatment reached a significant level. The seed setting rate was significantly different between cytoplasms under the N1, N2, and N3 treatments. The difference in effective panicles between the cytoplasms under the N2 and N3 (2023) treatments reached a significant level. The two-year yield analysis demonstrated that the yield of JW-type rice increased first and then decreased with the increase in nitrogen application rate, and the highest was 8195.55 kg/hm^2^ under the N2 treatment. The W-type yield increased with the increase in nitrogen application rate.

## 4. Discussion

### 4.1. Effect of Different Forms of Nitrogen on Nitrogen Absorption Rate

The nitrogen uptake efficiency of different rice varieties all increased with the increase in nitrogen application level [26,27], and the utilization efficiency decreased with the increase in nitrogen fertilizer application [28]; additionally, the different forms of nitrogen sources had a significant effect on the growth and nitrogen uptake metabolism of rice [29]. Under the conditions of this experiment, the rate of nitrogen uptake by the two cytoplasmic rice root systems was also significantly increased with the increase in the concentration of different forms of nitrogen. Therefore, the factors affecting the rate of nitrogen uptake were mainly the effects of nitrogen concentration; additionally, there were significant differences in nitrogen uptake between different sterile cytoplasmic root systems.

Rice roots absorb mainly ammonium nitrogen, and compared with nitrate nitrogen, rice has a better compatibility for ammonium nitrogen [30]. The reason might be that the valence of the inner cell membrane is negative and ammonium nitrogen is positive. Rice roots more easily absorb ammonium nitrogen because ammonium nitrogen absorption requires less energy than nitrate nitrogen does [31,32]. However, the nitrate nitrogen content is far higher than ammonium nitrogen in soil under natural conditions. Additionally, rice roots exhibit O_2_ secretion and sometimes are in an aerobic status. Oxidation makes some nitrate nitrogen in the soil convert into ammonium nitrogen, which is absorbed by the rice root. Therefore, in this study, the nitrate nitrogen absorption rate was slightly larger than that of ammonia nitrogen in rice, which may be related to that of the absorption liquid; only nitrate and rice genotypes exist in the absorption liquid; and the metabolic capability of the root increases because of the increase in culture liquid ventilation [33,34]. At the concentrations of this experiment, under the nitrogen uptake efficiency using different forms of nitrogen, the root nitrogen uptake of both types of cytoplasmic rice showed a linear increase trend with the increase in nitrogen concentration. At low concentrations, the absorption efficiency of ammonium nitrogen by the roots was slightly higher than that of nitrate nitrogen. At medium-to-high concentrations, the absorption rate of nitrate nitrogen was slightly higher than that of ammonia nitrogen, which may be due to the differences in the absorption mechanism of the roots under different nitrogen concentrations. This may be because the root system has different nitrogen concentration absorption mechanisms. When the nitrogen concentration is low, the ammonium (nitrogen) high-affinity transport system is in the dominant role; when the external nitrogen concentration is high, the ammonium (nitrogen) low-affinity transport system plays a dominant role [33,34]; in the low concentration of ammonium nitrogen, the root system shows greater affinity and transport capacity, as well as absorption, and less energy consumption, so the ammonium nitrogen uptake efficiency at a low concentration is higher than that of nitrate nitrogen.

### 4.2. The Effect of Different Cytoplasms on Nitrogen Absorption Rate

Rice is a crop adapted to ammonium and absorbs more ammonium nitrogen during the growth period. Whether under the condition of ammonium nitrogen or ammonium nitrogen, significant differences were observed among different genotypes and different concentrations of solution. The effect of genotypes interacting with the concentration of the solution had highly significant differences from each other for the nitrogen absorption rate. Under mixed nitrogen conditions, both cytoplasmic MS lines (WA803A and JW803A) showed identical nitrogen uptake trends. The effect of cytoplasmic WA was more complex for the regulation of ammonium nitrogen absorption by the nuclear gene. The JW cytoplasm exhibited additive effects in nuclear gene-mediated ammonium absorption at low concentrations and surpassed WA across all concentrations. The results showed that the JW cytoplasm exhibited greater ammonium uptake than WA, although both showed reduced ammonium nitrate absorption under nitrate conditions. The difference in absorption efficiency between WA803A and JW803A was not significant and was almost smaller than that of maintainer line 803B.

### 4.3. Effect of Cytoplasmic MS on Accumulation of Dry Matter and Nitrogen

Rice is a crop adapted to ammonium and absorbs more ammonium nitrogen during the growth period. However, the fertilizer nitrogen and NH_4_^+^, which is released from the mineralization of organic nitrogen in soil, are easily oxidated into NO_3_^−^ under good aeration conditions [35]. Significant differences were observed in the accumulation of dry matter and nitrogen between different genotypes at the heading stage. Under different nitrogen levels, the trends in dry matter and nitrogen accumulation for the two different cytoplasmic male sterile lines, WA803A and JW803A, were consistent, with both root dry matter and nitrogen accumulation decreasing with increasing nitrogen application. Under the N0 and N1 conditions, the WA cytoplasm accumulated more nitrogen than JW, and under the N2 and N3 conditions, the JW cytoplasm accumulated more dry matter and nitrogen than WA. This indicates that the JW and WA cytoplasms have different nitrogen absorption abilities at different nitrogen concentrations. Under low nitrogen conditions, the WA cytoplasm has a stronger nitrogen absorption ability than the JW cytoplasm, and under higher nitrogen concentrations, the JW cytoplasm has a stronger nitrogen absorption ability than the WA cytoplasm. Therefore, according to this research, cytoplasmic JW will show its superiority to cytoplasmic WA when the former is cultivated in winter paddy fields with bad aeration conditions and a low content of nitrogen. This result was tested under the condition of a single form of nitrogen but under the condition of both nitrate nitrogen and ammonium nitrogen existing. Further research is necessary to confirm the effect of different cytoplasms on the absorption rate of nitrogen.

## 5. Conclusions

The JW cytoplasm’s nitrogen uptake efficiency was significantly higher than that of the WA cytoplasm. In low concentrations of ammonium nitrogen, the JW cytoplasm showed the additive effect of nitrogen uptake with the same nuclei as the original maintainer line. The yield of JW-type rice increased first and then decreased with the increase in nitrogen application rate; the highest yield was 8195.55 kg/hm^2^ under the N2 treatment.

## Figures and Tables

**Table 1:** Analysis of variance of nitrogen absorption efficiency under different treatments.

Difference		SS	df	MS	F
Nitrate nitrogen	Genotype	703.91	2	351.95	626.77 **
	Solution concentration	37,469.47	8	4683.68	8340.82 **
	Interaction	1166.67	16	72.92	129.85 **
	Error	30.32	54	0.56	
Ammonium nitrogen	Genotype	324.29	2	162.14	658.49 **
	Solution concentration	12,913.47	7	1844.78	7491.93 **
	Interaction	618.00	14	44.14	179.27 **
	Error	11.82	48	0.25	

Note: “**” shows significant difference at the 0.01 level; each value represents the average of three repetitions of the test.

**Table 2:** Ammonium nitrogen absorption efficiency of different cytoplasm types.

Concentration of Ammonium Nitrogen (mmol/L)	WA803A		JW803A		803B
	Absorption Efficiency (umol/(g × h))	Compared with 803B (±%)	Absorption Efficiency (umol/(g × h))	Compared with 803B (±%)	Absorption Efficiency (umol/(g × h))
0.05	0.46	−18.67	2.17	279.41	0.57
0.10	2.21	256.39	4.75	666.17	0.62
0.20	3.43	353.50	4.86	542.54	0.76
0.40	6.58	94.47	9.83	190.33	3.39
0.60	9.01	−5.65	14.09	47.58	9.55
1.00	15.72	−8.47	24.35	41.72	17.18
1.40	25.89	11.30	34.10	46.58	23.26
1.80	32.33	−35.03	41.29	-17.03	49.76

Note: each value represents the average of three repetitions of the test.

**Table 3:** Nitrate nitrogen absorption efficiency of different cytoplasm types.

Concentration of Nitrate Nitrogen (mmol/L)	WA803A		JW803A		803B
	Absorption Efficiency (umol/(g × h))	Compared with 803B (±%)	Absorption Efficiency (umol/(g × h))	Compared with 803B (±%)	Absorption Efficiency (umol/(g × h))
0.05	1.03	−16.29	1.00	−19.04	1.23
0.1	1.96	7.19	1.85	0.97	1.83
0.2	2.01	−34.04	2.55	−16.38	3.05
0.4	6.54	−30.42	6.37	−32.24	9.40
0.6	15.57	−25.92	15.20	−27.67	21.01
1	23.78	−26.39	23.59	−26.97	32.30
1.4	29.67	−21.23	31.58	−16.15	37.67
1.8	48.51	−10.82	46.54	−14.46	54.40

Note: each value represents the average of three repetitions of the test.

**Table 4:** Response of sterile cytoplasm to nitrogen fertilizer in material output.

		2022				2023			
Treatment		Nutrient Organ Material Output Rate (%)	Nutrient Organ Material Conversion Rate (%)	Stem/Sheath Material Output Rate (%)	Stem/Sheath Matter Conversion Rate (%)	Nutrient Organ Material Output Rate (%)	Nutrient Organ Material Conversion Rate (%)	Stem/Sheath Material Output Rate (%)	Stem/Sheath Matter Conversion Rate (%)
JW	N0	42.44a	47.89a	35.99b	27.13b	31.63a	32.78a	33.27a	26.15a
	N1	31.98b	36.55b	29.82d	24.08c	28.26b	32.40a	26.09b	21.60b
	N2	14.53e	13.92e	7.36h	4.90f	10.51ab	10.56d	10.77e	8.00f
	N3	32.91b	35.41b	30.73c	23.80c	23.13c	24.84c	17.55d	12.94e
W	N0	25.97c	29.60c	21.76f	17.57d	29.41b	28.42b	31.11a	22.47c
	N1	31.13b	36.22b	27.01e	22.32cd	19.12d	22.04c	21.17c	18.24d
	N2	20.72d	21.88d	19.34g	15.00e	9.07f	8.89e	11.16e	8.11f
	N3	39.56ab	48.82a	39.58a	33.40a	15.63e	16.10d	11.77e	8.60f
F	M	90.70 **	56.37 **	80.63 **	37.31 **	59.59 **	62.62 **	47.50 **	52.31 **
	N	40.01 **	37.07 **	14.67 *	14.87 *	12.95 *	13.64 *	18.96 **	20.02 **
	M × N	87.56 **	72.94 **	39.47 *	39.66 *	111.22 **	112.34 **	28.45 *	42.20 **

Note: “*” shows the significant difference at the 0.05 level; “**” shows significant difference at the 0.01 level; each value represents the average of three repetitions of the test. Different letters in the same column indicate significant data differences (*p* < 0.05); JW: JW803A; W: WA803A.

**Table 5:** Differences in rice yield traits under different nitrogen fertilizer levels.

Year	Material	Variety	Spikelets per Panicle (×10^4^/hm^2^)	Spikelets per Panicle	Grain Filling (%)	1000-Grain Weight (g)	Grain Yield (kg/hm^2^)
2022	JW	N0	132.70a	157.78c	92.90a	28.77b	5593.17g
		N1	171.60a	164.49b	94.04a	29.73b	7907.5cd
		N2	175.22a	169.75b	96.06a	30.05b	8585.79b
		N3	188.41a	165.12c	91.66b	29.68a	8462.99b
	W	N0	133.25a	153.22c	93.61a	28.42b	5431.61gh
		N1	165.60a	157.50c	92.45b	30.76a	7417.10de
		N2	168.01a	171.63b	93.43b	30.38ab	8184.69c
		N3	184.81a	173.52b	93.69a	29.72a	8929.29a
2023	JW	N0	102.02a	181.07a	96.48a	29.28b	5216.47h
		N1	160.80ab	150.26c	94.88a	31.24b	7161.68f
		N2	162.03a	166.01b	95.37a	30.43b	7805.31d
		N3	146.40c	166.75b	94.00b	32.16a	7380.34e
	W	N0	108.10a	167.88b	94.62b	29.92ab	5132.95h
		N1	170.42a	159.64b	95.31a	32.35a	8372.37bc
		N2	150.06bc	170.12ab	95.27a	32.29a	7852.23d
		N3	171.62a	168.89b	94.51a	31.91a	8708.01ab

Note: Different letters in the same column indicate significant data differences (*p* < 0.05); each value represents the average of three repetitions of the test. JW: JW803A; W:WA803A.

## Data Availability

The data presented in this study are available upon request from the authors.
